# Long-Term Follow-Up of a Child with Autoimmune Thyroiditis and Recurrent Hyperthyroidism in the Absence of TSH Receptor Antibodies

**DOI:** 10.1155/2014/749576

**Published:** 2014-07-09

**Authors:** Christopher Dunne, Francesco De Luca

**Affiliations:** Section of Endocrinology and Diabetes, St. Christopher's Hospital for Children, Drexel University College of Medicine, 3601 A Street, Philadelphia, PA 19134, USA

## Abstract

Hashitoxicosis is an initial, transient, hyperthyroid phase that rarely affects patients with Hashimoto thyroiditis. We present here an unusual case of a child with Hashimoto thyroiditis and recurrent hyperthyroidism. A 4 yr 6/12 old male was diagnosed by us with autoimmune subclinical hypothyroidism (normal free T4, slightly elevated TSH, and elevated TG antibody titer). Two years and 6/12 later he experienced increased appetite and poor weight gain; a laboratory evaluation revealed suppressed TSH, elevated free T4, and normal TSI titer. In addition, an I^123^ thyroid uptake was borderline-low. A month later, the free T4 had normalized. After remaining asymptomatic for 3 years, the patient presented again with increased appetite, and he was found with low TSH and high free T4. Within the following 3 months, his free T4 and TSH normalized. At his most recent evaluation, his TSH was normal and the free T4 was borderline-high; the TG antibody titer was still elevated and the TSI titer was negative. To our knowledge, this is the first patient reported with Hashimoto thyroiditis and recurrent hyperthyroidism. This case exemplifies the variability of the manifestations and natural history of Hashimoto thyroiditis and supports the need for a long-term evaluation of patients with autoimmune thyroid disease.

## 1. Introduction

Hashitoxicosis is considered to be an initial hyperthyroid phase of Hashimoto thyroiditis, caused by the release of preformed thyroid hormones from the thyroid follicles. It is biochemically characterized by elevated titer(s) of anti-thyroglobulin (anti-TG) and/or anti-thyroid peroxidase (anti-TPO) antibodies, suppressed TSH, and elevated T4 and T3. Hashitoxicosis is distinguished from Graves disease by the absence of thyroid stimulating immunoglobulins (TSI) and by a diminished radioiodine uptake. We present an unusual case of recurrent hyperthyroidism in a child with positive anti-thyroglobulin antibodies and negative TSI. This case illustrates the need for continued follow-up of patients with autoimmune thyroid disease and supports the evidence of a very wide spectrum of clinical and biochemical features in children with autoimmune thyroid disease.

## 2. Case Presentation

A 4 yr 6/12 old male was first seen at our clinic in 2006, after his primary physician found him with a slightly elevated TSH (Table 1) approximately 1 month prior. The review of systems was contributory only for mild constipation. His past medical history was significant for speech delay, asthma, and ADHD (diagnosed 3 weeks earlier). Upon his initial evaluation at our clinic, his weight and height were at the 50th and 25th percentiles, respectively. He was found with a normal thyroid size and no palpable thyroid nodules or regional lymphadenopathy. His family history for thyroid disorders was negative. His repeated TSH was still mildly elevated, while the free T4 was normal (Table 1); anti-TG antibodies were positive and anti-TPO antibodies were negative (Table 1). A month later, a thyroid ultrasound demonstrated normal size, contour, and echo texture, without mass or nodules. Based on the elevated TSH, the patient was placed on L-thyroxine (50 micrograms daily).

In the following year, the patient was maintained on the same dose of L-thyroxine, but he continued to experience hyperactivity, poor appetite, and speech delay. In addition to L-thyroxine, the patient was placed on Atomoxetine (40 mg QD) for ADHD. At age 5 yrs 7/12 (13 months after the initial visit), the patient and his mother returned to our clinic for follow-up with complaints of insomnia, aggressiveness, heat intolerance, and persistent constipation. His mother admitted being nonadherent to the recommended treatment, giving her son L-thyroxine only 3-4 times a week. Height and weight had remained within normal limits, and his thyroid size was normal. A month prior to the follow-up visit, the TSH was 4.49 mIU/dL (normal range, 0.5–4.3) and the free T4 1.05 ng/dL (normal range, 0.9–1.4). At the end of the follow-up visit, we advised his mother to discontinue the L-thyroxine treatment and repeat the thyroid hormone measurements 6 weeks later; however, this laboratory evaluation was never obtained.

About a year after the follow-up visit, the patient, now 6 yr 8/12 old, was brought again to our clinic. Meanwhile, he had been placed in special education due to his developmental delay and behavioral difficulties; Risperidone was added to his treatment regimen (still taking Atomoxetine). The child complained of occasional headaches but was otherwise asymptomatic. His weight and height remained at the 50th percentile, and he was found to be clinically and biochemically euthyroid (Table 1). Thus, his mother was told that no further endocrine evaluation was necessary.

Approximately four months later, he was referred back to us by his primary physician. Due to worsening hyperactivity, increased appetite, and poor weight gain (1 kg in 4 months), he requested thyroid function tests. The child's TSH was suppressed (<0.01 mIU/L) and the free T4 elevated (2.8 ng/dL), with a normal TSI of 90% (normal, <140%). Anti-TPO antibodies remained undetectable, while the anti-TG antibodies were elevated (Table 1). Based on these findings, we requested an I^123^ thyroid uptake, which was borderline-low (7.3% at 4 hours, with a normal range of 5–15%; 11.7% at 24 hours, with a normal range of 10–40%); therefore, the diagnosis of Hashitoxicosis was made. Anticipating a short-lived hyperthyroid phase, we repeated the thyroid function tests a month later. At that time, his free T4 (1.2 ng/dL) and total T3 (128 ng/dL: normal range, 94–213) were both normal; he was also reported to be less hyperactive. As a result, no anti-thyroid medication was initiated.

At the age of 7 yr 10/12 (3 yrs 4/12 after the initial diagnosis of autoimmune thyroid disease and 11/12 after his short-lived hyperthyroidism), he presented to our clinic with complaints of constipation and occasional headaches. Upon examination in our clinic, his thyroid was not palpable and his thyroid hormone levels were normal (Table 1). During the following 2 years, the patient remained clinically and biochemically euthyroid while completely off thyroid medication (Table 1).

At 9 yr 10/12 he was again noted by his mother to be more hyperactive, with increased appetite and thirst. When evaluated by us, he was found with a normal thyroid size, shape, and contour. An ultrasound revealed heterogeneous appearance of the thyroid and confirmed its normal size; no discrete nodules were identified. The TSH was low and the free T4 was high (Table 1). In addition, the TG antibody titer was high, while the TPO and the TSI titers were negative (Table 1). In light of his prior history of transient hyperthyroidism, we chose to wait and not treat. A month later, he was found with a normal free T4 (1.0 ng/dL), a higher TSH (0.24 mIU/L), low total T3 (77 ng/dL), elevated TG antibodies (399 IU/mL: normal, <20), and negative TPO antibodies. Three months later, the TSH rose to 6.32 mIU/L and his free T4 was still normal at 1.1 ng/dL.

At his most recent visit at our center (11 yr 11/12 of age; 6 yrs 5/12 after his initial presentation), his TSH and total T3 were normal, while the free T4 was borderline-high (Table 1); the TG antibody titer persisted to be elevated and the TPO titer negative.

## 3. Discussion

Chronic autoimmune thyroiditis is the most common thyroid disorder in children. Also known as Hashimoto thyroiditis, it is typically characterized by elevated titers of thyroid autoantibodies. Thyroid peroxidase (TPO) antibodies are detectable in ~95% of patients with Hashimoto's thyroiditis, and thyroglobulin TG antibodies are positive in approximately 60% of adult patients with chronic thyroiditis (less often in children with thyroid autoimmune disease). Clinically and biochemically, chronic thyroiditis is characterized by a variable presentation in childhood: children of younger age are most often symptomatic [1]. Affected children may present with normal thyroid hormone levels, subclinical hypothyroidism, or overt hypothyroidism. A small percentage of patients who are initially euthyroid may persistently remain euthyroid, while a few patients with subclinical hypothyroidism may revert to a euthyroid state. Very rarely at diagnosis, they may exhibit short-lived signs and symptoms of hyperthyroidism, which tend to resolve in a few weeks and be followed by a euthyroid or hypothyroid state [2]. The initial hyperthyroid phase in chronic autoimmune thyroiditis, also known as “Hashitoxicosis,” is due to leakage of preformed hormones from the inflamed thyroid gland [3].

The clinical history and laboratory findings of our patient are surely atypical for a child with thyroid disease. First, unlike most children with chronic thyroiditis, the only thyroid autoantibodies that have been persistently positive are the anti-thyroglobulin antibodies. Furthermore, the patient has experienced recurrent hyperthyroidism, preceded and followed by years of normal thyroid function. Although some psychotropic medications (like lithium) are sometimes associated with impaired thyroid function, Risperidone and Atomoxetine are not typically implicated in drug-related thyroid dysfunction. Lastly, some of the symptoms experienced by this patient, such as his behavioural difficulties, are often associated with a hyperthyroid state; however, in this case they also occurred when the patient was biochemically euthyroid.

There is now genetic [4] as well as pathophysiological [5] evidence that Graves disease and Hashimoto thyroiditis may be different manifestations of one continuous spectrum of autoimmune thyroid disease. In both Graves disease and Hashimoto thyroiditis, thyroid-reactive T lymphocytes are formed and infiltrate the thyroid gland [6]. In Graves disease, T Helper 2 lymphocytes stimulate the TSH-Receptor antibody production by B lymphocytes. In Hashimoto, T Helper 1 lymphocytes infiltrating the thyroid induce apoptosis of thyroid follicular cells, often generating hypothyroidism [5]. Abnormal regulatory T cells may be the common denominator found in both disorders; they are abundantly found in inflamed thyroid tissue, and in disease states, they seem to be unable to downmodulate the autoimmune response [5]. Subjects with Graves disease often exhibit detectable TPO and/or TG antibodies (which are then nonspecific markers of Hashimoto thyroiditis), although the most commonly detectable thyroid autoantibodies in autoimmune hyperthyroidism are TSI. Clinical and biochemical shift from Graves disease to Hashimoto hypothyroidism has been widely described, and although less frequently reported, Graves disease may occur after the diagnosis of Hashimoto's [7–9]. Takasu et al. [7] described seven adult patients with autoimmune hypothyroidism who developed Graves disease (hyperthyroidism with positive thyroid stimulating antibodies). In some of these patients hyperthyroidism was transient and followed by hypothyroidism, while in others it was persistent. However, none of the seven patients experienced recurrent hyperthyroidism. In addition, the authors did not comment on whether the thyroid stimulating antibodies were measured at the onset of hypothyroidism; therefore we do not know whether there was a true conversion to elevated TSI titer associated with the change in thyroid function.

It has also been reported that Graves disease is sometimes associated with negative TSI [10–12].Rahhal and Eugster [10] describe fifty-seven pediatric patients (mean age 11.5 years) with clinical and biochemical signs of hyperthyroidism: twenty-six of the fifty-seven children were found with negative TSI. Many patients (forty-seven of these fifty-seven children) underwent I^123^ thyroid uptake scans, and all of them had elevated uptake, thus suggesting the diagnosis of Graves disease. The authors concluded that a negative TSI titer “should not introduce diagnostic uncertainty in the setting of classic clinical and biochemical features”; however, one wonders how many of the patients with negative TSI had an elevated uptake. Moreover, since the antibodies were only measured at diagnosis it is possible that some of the TSI-negative patients with an elevated uptake may have developed a positive TSI titer after the initial presentation.

Our patient's persistently negative TSI titer was associated with borderline-low radioiodine thyroid uptake, which argues against the diagnosis of Graves disease. Low thyroid autoantibody titers and hyperthyroidism may also occur in patients with subacute or viral thyroiditis; yet, in this thyroid disorder, markers of autoimmunity are usually transiently elevated, and signs and symptoms of hyperthyroidism present at diagnosis and do not recur years later. The short-lived occurrence of hyperthyroidism and reduced iodine uptake in our patient most resemble the typical presentation of Hashitoxicosis; on the other hand, we are not aware of any previous report of Hashitoxicosis in children that occurred long after the diagnosis of autoimmune thyroid disease and then recurred years later. Indeed, Wasniewska et al. [13] followed fourteen children with autoimmune thyroid disease, negative TSI and hyperthyroidism occurring within a few months from the initial diagnosis. After hyperthyroidism resolved, no relapses were recorded up to nine years after the diagnosis of autoimmune thyroiditis. In another study, Nabhan et al. [14] found eight out of sixty-nine children diagnosed with autoimmune thyroiditis who were initially hyperthyroid. Three of the eight children had elevated TSI, and (among the four patients who underwent ^123^I thyroid uptake) two children had increased uptake. Of note, none of the eight patients experienced a recurrence during the follow-up period.

In conclusion, our patient represents a further example of the variability of the clinical and biochemical manifestations of autoimmune thyroid disease in children. In addition, it confirms how little we know about the natural history and the outcome of children with chronic thyroiditis. As a result, we believe that a long-term, periodical evaluation of patients with autoimmune thyroid disease (despite the lack of clinical and biochemical manifestations of an overt thyroid dysfunction for many years) is always warranted.

## Figures and Tables

**Table 1:** Initial and follow-up laboratory studies.

Date	Age (yr)	Free T4 (0.9–1.4 ng/dL)	TSH (0.5–4.3 mIU/L)	T3 (94–213 ng/dL)	TPO (<35 IU/mL)	TG Ab (<20 IU/mL)	TSI (<140%)
07/17/2006	46/12	1.1	7.94	140			
08/24/2006	47/12	1.1	9.26		<10	85	
08/29/2007	57/12	1.05	4.49				
10/23/2008	68/12	1.39	3.21				
03/6/2009	71/12	2.8	<0.01		<10	312	90
04/30/2009	72/12	1.2		128	<10	279	107
12/2/2009	710/12	1.0	3.34	112	<10		96
06/16/2010	84/12	1.3	2.23	110	<10	61	100
12/28/2011	910/12	2.1	0.02				39
03/9/2012	101/12	1.0	0.24	77	12	399	
07/5/2012	104/12	1.1	6.32				
02/6/2014	1111/12	1.5	1.77	102	<10	59	43

Normal ranges in parentheses.

## Abbreviations

TPO: Thyroid peroxidase
TG: Thyroglobulin
TSI: Thyroid stimulating immunoglobulins.
